# Transgenic Dendra2::tau expression allows *in vivo* monitoring of tau proteostasis in *Caenorhabditis elegans*

**DOI:** 10.1242/dmm.050473

**Published:** 2024-03-28

**Authors:** Marina Han, Aleen Saxton, Heather Currey, Sarah M. Waldherr, Nicole F. Liachko, Brian C. Kraemer

**Affiliations:** ^1^ Graduate Program in Neuroscience, University of Washington, Seattle, WA 98195, USA; ^2^ Division of Gerontology and Geriatric Medicine, Department of Medicine, University of Washington, Seattle, WA 98104, USA; ^3^ Geriatrics Research Education and Clinical Center, Veterans Affairs Puget Sound Health Care System, Seattle, WA 98108, USA; ^4^ Department of Psychiatry and Behavioral Sciences, University of Washington, Seattle, WA 98195, USA; ^5^ Department of Laboratory Medicine and Pathology, University of Washington, Seattle, WA 98195, USA

**Keywords:** Tau, *Caenorhabditis elegans*, Alzheimer's disease, Proteostasis, Dendra2, TDP-43, TARDBP

## Abstract

Protein homeostasis is perturbed in aging-related neurodegenerative diseases called tauopathies, which are pathologically characterized by aggregation of the microtubule-associated protein tau (encoded by the human *MAPT* gene). Transgenic *Caenorhabditis elegans* serve as a powerful model organism to study tauopathy disease mechanisms, but moderating transgenic expression level has proven problematic. To study neuronal tau proteostasis, we generated a suite of transgenic strains expressing low, medium or high levels of Dendra2::tau fusion proteins by comparing integrated multicopy transgene arrays with single-copy safe-harbor locus strains generated by recombinase-mediated cassette exchange. Multicopy Dendra2::tau strains exhibited expression level-dependent neuronal dysfunction that was modifiable by known genetic suppressors or an enhancer of tauopathy. Single-copy Dendra2::tau strains lacked distinguishable phenotypes on their own but enabled detection of enhancer-driven neuronal dysfunction. We used multicopy Dendra2::tau strains in optical pulse-chase experiments measuring tau turnover *in vivo* and found that Dendra2::tau turned over faster than the relatively stable Dendra2. Furthermore, Dendra2::tau turnover was dependent on the protein expression level and independent of co-expression with human TDP-43 (officially known as TARDBP), an aggregating protein interacting with pathological tau. We present Dendra2::tau transgenic *C. elegans* as a novel tool for investigating molecular mechanisms of tau proteostasis.

## Teaser

**Summary:** Diseases exhibiting pathological tau protein constitute the majority of dementia cases. Investigation of molecular tauopathy mechanisms in *Caenorhabditis elegans* strains expressing photoconvertible Dendra2::tau can inform disease mechanisms.

## INTRODUCTION

Humans become more susceptible to neurodegenerative disease with age in part due to the stress of maintaining neuronal health across a longer lifespan and the accumulation of pathological proteins (Hetz, 2021). Tauopathies are a group of age-related neurodegenerative disorders caused by pathological hyperphosphorylation and aggregation of the microtubule-binding protein tau (encoded by *MAPT*). They are clinically characterized by various manifestations of behavioral, motor, language and memory impairments. Of the many distinct tauopathies, Alzheimer's disease (AD) accounts for 60-70% of dementia, whereas frontotemporal lobar degeneration accounts for 2.6% (Zhang et al., 2022), making the treatment of these diseases a priority for dementia and aging research. Furthermore, although AD is also characterized by the accumulation of amyloid-β peptides, tau burden and not amyloid-β pathology has been shown to correlate with disease severity (Nelson et al., 2012), indicating that tau-targeting therapies may be valuable in ameliorating clinical progression.

In healthy cellular physiology, tau binds to microtubules to promote their assembly and stability, thereby enabling axonal transport. A small fraction of tau localizes to dendrites, dendritic spines and the post-synapse, where tau plays a yet undefined role in healthy synaptic function. Indeed, loss of tau leads to synaptic defects and functional impairment, whereas tau hyperphosphorylation and aggregation compromise short- and long-term plasticity (Ittner and Ittner, 2018). Tau also plays a role in myelination, neurogenesis, iron homeostasis, glucose metabolism and DNA protection (Wang and Mandelkow, 2016; Kent et al., 2020). As a protein that is highly expressed in neurons, proper localization and function of tau is integral to neuronal health.

In disease, hyperphosphorylated tau dissociates from microtubules to form oligomers and eventually accumulates as insoluble fibrils, disrupting neuronal function. Tau deposits exist mostly in the cytoplasm of neuronal and glial processes but can also be found in the nucleus. Tau can bind to RNA to form complexes in both cellular compartments, mislocalizing nuclear speckle components and disrupting microtubule dynamics (Ginsberg et al., 1997; Lester et al., 2021; McMillan et al., 2023). In primary tauopathies such as frontotemporal lobar degeneration, Pick's disease, corticobasal degeneration and progressive supranuclear palsy, mutations in *MAPT* cause changes in expressed tau splice isoforms, post-translational modifications, microtubule affinity, folding or aggregation propensity, which likely lead to the various pathological tau structures and localization observed in these diseases (Naseri et al., 2019; Strang et al., 2019; Gallo et al., 2022). In contrast, most cases of AD are sporadic with wild-type (WT) tau forming aggregates. How tau becomes pathological remains unclear and, consequently, tauopathies remain untreatable. Tau levels can be decreased by inhibiting tau production or enhancing tau degradation. The latter can be achieved by targeting mechanisms of tau protein homeostasis (proteostasis).

Normally, cellular proteostasis mechanisms activate in response to an overabundance or misfolding of protein. These mechanisms include the autophagy lysosomal pathway (ALP), ubiquitin–proteasome system (UPS) and the unfolded protein response (UPR). The ALP degrades cytosolic and membrane-enclosed proteins, particularly larger ones such as those that form aggregates and those within organelles, through engulfment by phagophores that fuse with lysosomes. The UPS selectively degrades soluble proteins that have been tagged with ubiquitin. Misfolded protein accumulation in the endoplasmic reticulum (ER) or mitochondria activates the UPR, resulting in transcriptional activation of proteostasis genes. In the case of the ER UPR (or UPR^ER^), ER-associated degradation directs misfolded proteins to the proteasome but can also activate autophagy.

Proteostasis impairment is a hallmark of aging (López-Otín et al., 2013; Kennedy et al., 2014). Accumulation of pathological tau further compromises neuronal proteostasis mechanisms. Full-length tau is selectively degraded by the UPS, whereas truncated, aggregated or soluble mutant tau is degraded by the ALP (Wang et al., 2009; Dolan and Johnson, 2010; Wang and Mandelkow, 2012; Hamano et al., 2021). However, tau accumulation impairs autophagosome–lysosome fusion by inhibiting expression of IST1, a positive modulator of the endosomal sorting complex required for transport (ESCRT) machinery (Feng et al., 2020). In addition, acetylated tau inhibits chaperone-mediated autophagy, rerouting tau to be degraded by macroautophagy and endosomal microautophagy (Caballero et al., 2021). Impairing autophagy by various mechanisms leads to increased tau secretion, enabling cell-to-cell seeding (Tang et al., 2015; Chen et al., 2020; Caballero et al., 2021). Consequently, activating autophagy with IST1 upregulation, trehalose, methylene blue or mammalian target of rapamycin (mTOR) inhibitors results in reduction of tau levels *in vivo* and *in vitro* (Schaeffer et al., 2012; Hamano et al., 2021).

Abnormal levels and mislocalization of multiple UPS components correspond with phosphorylated and ubiquitinated tau pathology in human AD brains (Weng and He, 2021). For instance, AD brains exhibit increased levels of the carboxyl terminus of Hsp70-interacting protein (CHIP or STUB1) (Sahara et al., 2005), the E3 ubiquitin ligase that ubiquitinates tau paired helical filaments, targeting it to the 26S proteasome (Mori et al., 1987; Petrucelli et al., 2004). Interestingly, deletion of CHIP increases tau accumulation but not aggregation (Dickey et al., 2006), likely because CHIP-mediated hyper-ubiquitination of phosphorylated tau promotes its aggregation (Petrucelli et al., 2004; Kim et al., 2021). Despite its ubiquitination, the proteasome fails to degrade phosphorylated or insoluble tau. Tau becomes a ubiquitin sink, blocking ubiquitin recycling and potentially obstructing the proteasome core particle (Weng and He, 2021). Rescuing proteasome function with a proteolysis targeting chimera (PROTAC) improves tau clearance and phenotype in AD and tauopathy mouse models (Wang et al., 2021).

The relationship between tau and the UPR^ER^ remains controversial. Disease-specific brain regions of AD, progressive supranuclear palsy, and Pick's disease brains exhibit abnormally activated UPR^ER^ (Hoozemans et al., 2005, 2009; Nijholt et al., 2012; Stutzbach et al., 2013). Tau accumulation activates the UPR^ER^ *in vivo* by compromising ER-associated degradation (Abisambra et al., 2013), and conversely, ER stress results in increased tau phosphorylation (Ho et al., 2012). Multiple studies have shown that UPR^ER^ activation protects against tau toxicity (Loewen and Feany, 2010; Bruch et al., 2017; Waldherr et al., 2019; Shin et al., 2021), but understanding the exact mechanism requires further investigation. Evidenced by the multitude of disease-associated alterations in proteostasis mechanisms, tau pathology is deeply intertwined with the neuronal proteostatic network. Therefore, studying tau proteostasis is imperative for developing effective tau-targeting therapies.

We used transgenic *Caenorhabditis elegans* to investigate molecular mechanisms of tau proteostasis. Powerful genetic tools, short lifespan, ease of imaging, a thoroughly documented connectome and high-throughput functional assays make *C. elegans* useful for studying age-related neurodegenerative tauopathy. Transgenic *C. elegans* tauopathy models exhibit quantifiable neurological deficits mirroring the molecular and cellular features of human neuropathology: uncoordinated movement, neuronal loss, disease protein aggregation and shortened lifespan (Kraemer et al., 2003). However, most previous models do not capitalize on a key strength of the model system, which is live imaging of cellular function and protein trafficking. Furthermore, many *C. elegans* neurodegenerative disease models utilize multicopy transgenes yielding high levels of disease protein overexpression to elicit a phenotype that presents limitations in the precision of the transgenic modelling strategy.

Here, we characterize a suite of tau transgenic strains addressing both shortcomings. We used conventional transgene arrays and recombinase-mediated cassette exchange to generate several multicopy and single-copy genomically integrated strains pan-neuronally expressing the photoconvertible protein Dendra2 fused to WT human tau (Dendra2::tau) as a system for monitoring tau proteostasis. Upon exposure to 405 nm light, Dendra2 irreversibly converts from green to red fluorescence. Dendra2 possesses several advantages over other photoactivatable fluorescent proteins: monomeric form, high-contrast photoconversion, high photostability, bright fluorescence and low phototoxic activation (Chudakov et al., 2007). The Dendra2::tau model is a useful system for studying tau proteostasis because it allows immediate visualization of tau localization and accumulation and enables optical pulse-chase experiments to measure tau turnover *in vivo*. As approximately 60-80% of *C. elegans* genes have an analogous human counterpart and about 42% of human disease genes have a *C. elegans* ortholog (Markaki and Tavernarakis, 2010), genetic targeting of tauopathy disease mechanisms in Dendra2::tau *C. elegans* could shed light on how those mechanisms affect tau proteostasis and could be translatable to mammalian models.

## RESULTS

### Multicopy Dendra2::tau *C. elegans* exhibit a range of disease phenotypes

**Fig. 1.**
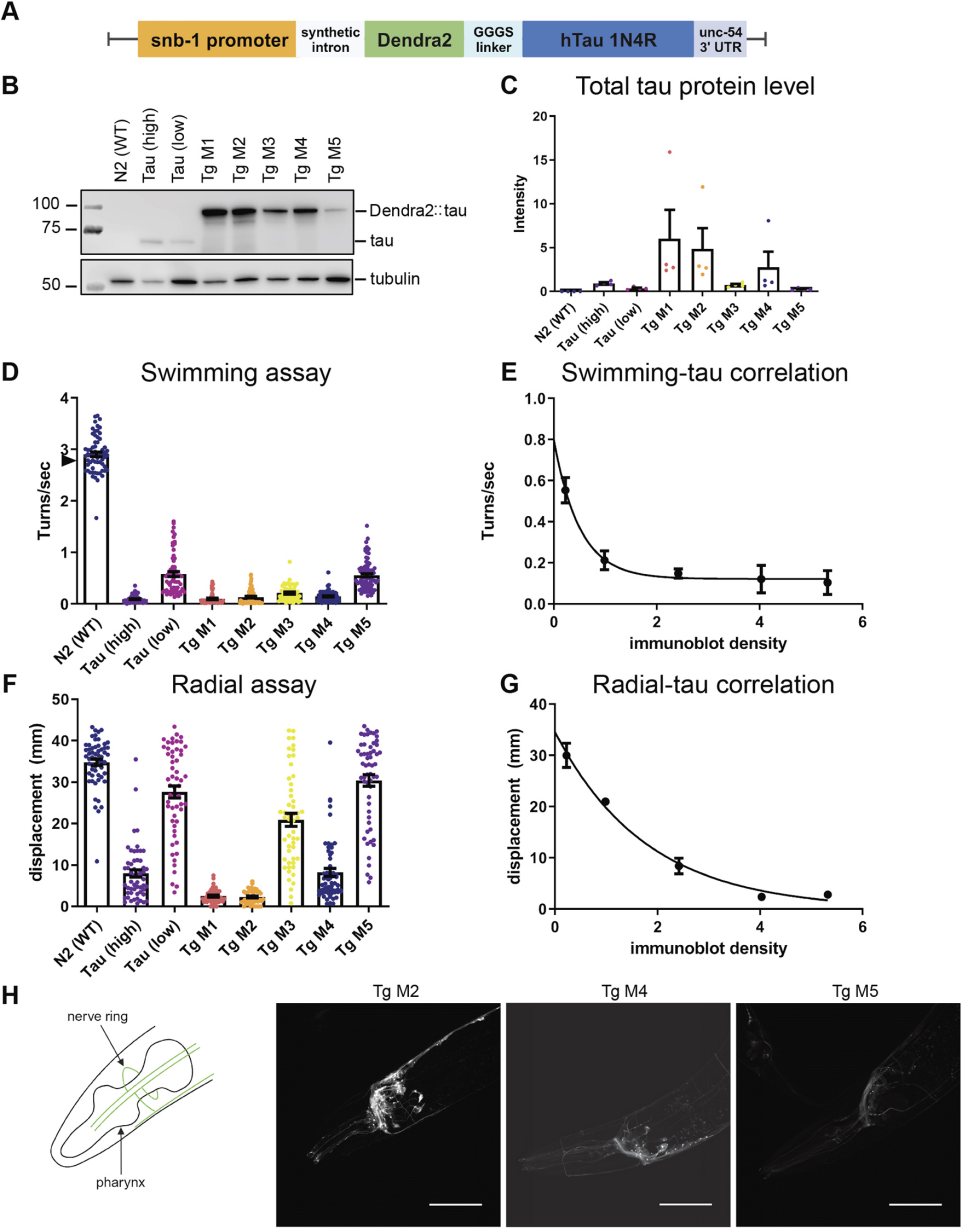
**Multicopy Dendra2::tau levels correlate with behavioral phenotype.** (A) DNA construct used for multicopy genomic integration of Dendra2::tau by ultraviolet irradiation. Note that the Dendra2 construct is driven by the *snb-1* promoter, not the previously published *aex-3* promoter for untagged tau strains. (B) Western blot detecting total tau protein. (C) Western blot quantification of total tau band intensity in untagged and Dendra2-tagged tau strains normalized to tubulin band intensity [four biological replicates; one-way ANOVA with Tukey's multiple comparison test (*P*=0.0493)]. (D) Swimming assay [three biological replicates; *n*≥49 animals/strain; one-way ANOVA with Tukey's multiple comparison test (*P*<0.0001)]. The arrowhead indicates the average performance of Dendra2-only control strain Tg 2 (Fig. S1). (E) Dendra2::tau *C. elegans* swimming behavior correlates with total tau protein levels following an exponential decay relationship (R^2^=0.8247). (F) 24 h radial assay [four biological replicates; *n*≥48 animals/strain; one-way ANOVA with Tukey's multiple comparison test (*P*<0.0001)]. (G) Dendra2::tau *C. elegans* radial locomotion correlates with total tau protein levels following an exponential decay relationship (R^2^=0.9513). (H) Schematic (left) and representative confocal images (right) of the head of three multicopy Dendra2::tau strains. Scale bars: 50 μm. Data are mean±s.e.m.

We generated five independent pan-neuronal Dendra2::tau-expressing transgenic (Tg) *C. elegans* strains using genomically integrated multicopy arrays (Tg M1-M5) under control of the pan-neuronal *snb-1* promoter (Fig. 1A). Dendra2::tau Tg animals were characterized for their tau protein level, behavior and Dendra2 fluorescence. Dendra2::tau protein expression and behavioral deficits were compared to previously published strains with high and low expression of untagged tau [Tau (high) and Tau (low), respectively] (Kraemer et al., 2003; Benbow et al., 2020), which both express WT human tau under the pan-neuronal *aex-3* promoter. The five Dendra2::tau transgenic strains exhibited a broad range of tau protein expression (Fig. 1B,C) correlating with motility impairment (Fig. 1D,F), with higher tau levels corresponding to greater motility deficits in a manner consistent with an exponential decay relationship (Fig. 1E,G). To better differentiate the levels of motility impairment between the Tau (high), Tg M1 and Tg M2 strains, we performed a radial assay that showed that Tg M1 and Tg M2 dispersed significantly less over a 24 h period compared to Tau (high) (Fig. 1F), as would be expected from the higher burden of tau in these strains. Therefore, we attribute the lack of a significant difference between the Tg M1, Tg M2 and Tau (high) strains in the swimming assay to a floor effect in the motor program measured by this assay. Confocal microscopy confirmed that the Dendra2 construct was indeed expressed in a neuronal pattern (Fig. 1H). By qualitative observation, the Dendra2 fluorescence intensity of the multicopy strains corresponded to their relative tau protein levels. In summary, we generated a collection of Dendra2::tau-expressing strains with a broad range of pan-neuronal tau expression levels driving increasingly severe behavioral phenotypes.

### Single-copy Dendra2::tau *C. elegans* display a WT phenotype

**Fig. 2.**
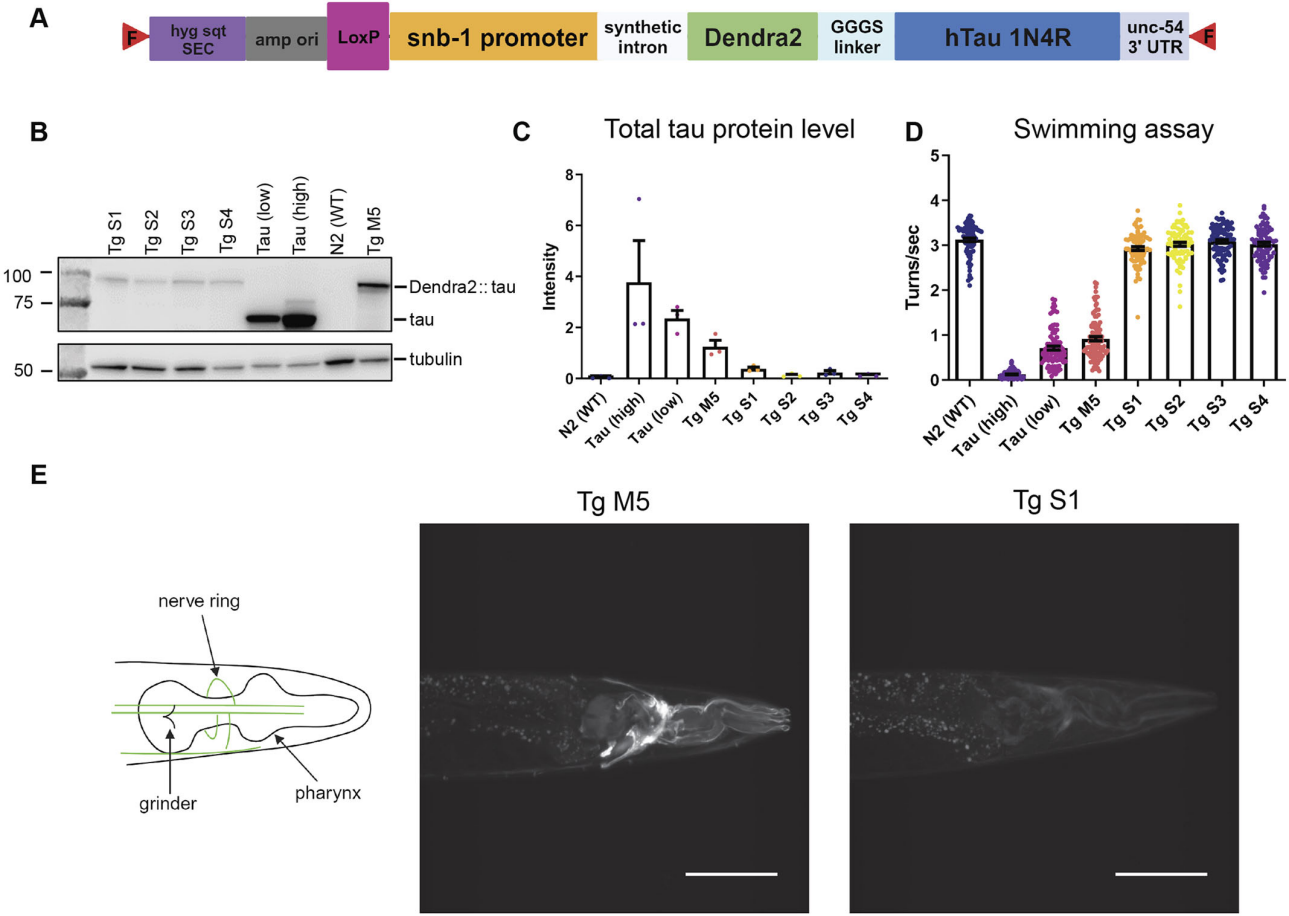
**Single-copy Dendra2::tau does not confer an obvious disease phenotype.** (A) DNA construct used for single-copy insertion of Dendra2::tau by dual-component recombinase-mediated cassette exchange in a miniMos landing site (Nonet, 2020). F, *FRT* site; *hyg sqt* SEC, a self-excising cassette consisting of a promoterless hygromycin-resistance gene, the *sqt-1(e1350)* gene, and *cre* recombinase under the control of the *hsp-16.1* promoter; amp, β-lactamase gene; ori, *E. coli* plasmid replication origin. (B) Western blot detecting total tau. (C) Western blot quantification of total tau band intensity in untagged and Dendra2-tagged tau strains normalized to tubulin band intensity [four biological replicates; one-way ANOVA with Tukey's multiple comparison test (*P*<0.0001)]. (D) Swimming assay [four biological replicates; *n*≥77 animals/strain; one-way ANOVA with Tukey's multiple comparison test (*P*<0.0001)]. (E) Schematic (left) and representative confocal image (right) of the lowest-expression Dendra2::tau multicopy strain (Tg M5) compared to a representative image of a single-copy Dendra2::tau strain (Tg S1). Scale bars: 50 μm. Data are mean±s.e.m.

Many *C. elegans* models of neurodegenerative disease utilize extrachromosomal arrays or integrated transgenes expressing a disease-related protein. To address concerns raised about the potential differences between multicopy versus single-copy transgenic strategies, we generated *C. elegans* strains with a single copy of genomically integrated Dendra2::tau (Tg S1-S4) using recombinase-mediated cassette exchange (Fig. 2A) (Nonet, 2020). When compared to untagged tau strains [Tau (high) and Tau (low)] and the multicopy strain with the lowest tau expression (Tg M5), all four of the single-copy Dendra2::tau strains exhibited very low levels of tau protein (Fig. 2B,C) and performed similarly to the WT N2 strain in the swimming assay (Fig. 2D). The low level of tau was reflected by a very low fluorescence signal of Dendra2::tau detected by confocal microscopy, compared to the lowest-expressing multicopy Dendra2::tau Tg M5 strain (Fig. 2E).

To determine the usefulness of this model, we co-expressed single-copy Dendra2::tau with the known tau enhancer, WT human transactive response DNA-binding protein (TDP-43; officially known as TARDBP) (Latimer et al., 2022). Dendra2::tau fluorescence quantitated from confocal microscopy images revealed increased fluorescence intensity when single-copy Dendra2::tau was co-expressed with TDP-43 (Fig. S2A,B). We also show that TDP-43 expression exacerbated motor impairment of single-copy Dendra2::tau-expressing animals at days 1 and 5 of adulthood (Fig. S2C,D), demonstrating the utility of the single-copy Dendra2::tau model in detecting tauopathy phenotype exacerbation.

### Dendra2::tau protein turnover is faster than that of Dendra2

The monomeric fluorescent protein Dendra2 irreversibly converts from green to red fluorescence upon exposure to 405 nm light. Photoconversion of Dendra2::tau facilitates optical pulse-chase experiments by enabling measurement of red fluorescence decay, which represents pulse-labeled tau degradation. Meanwhile, return of green fluorescence indicates synthesis of new tau protein.

Previous studies found that photoconverted Dendra2 is stable and does not decay significantly over 200 min in HEK293 cells (Zhang et al., 2007) or over 24 h in *C. elegans* (Hamer et al., 2010). In contrast, the red fluorescence signal of Dendra2 tagged to 1N4R tau [containing one 29-residue inserts in the N-terminal half (‘1N’) and four microtubule-binding repeats (‘4R’)] decays to ∼20% of original intensity after 36 h in zebrafish (Lopez et al., 2017). In contrast, Dendra2 and Dendra2::0N4R tau in mouse brain-slice culture exhibited similar half-lives of 2.47 and 2.67 days, respectively (Croft et al., 2021).

We generated control strains expressing the Dendra2 protein by itself under the same pan-neuronal *snb-1* promoter. Dendra2 is at most minimally toxic based on performance in a motility assay (Fig. S1). Of the multiple Dendra2-expressing strains generated, the strain with no quantifiable behavioral deficits and lowest Dendra2 fluorescence was chosen for this experiment to avoid toxicity from high Dendra2 transgene expression (Fig. S1).

**Fig. 3.**
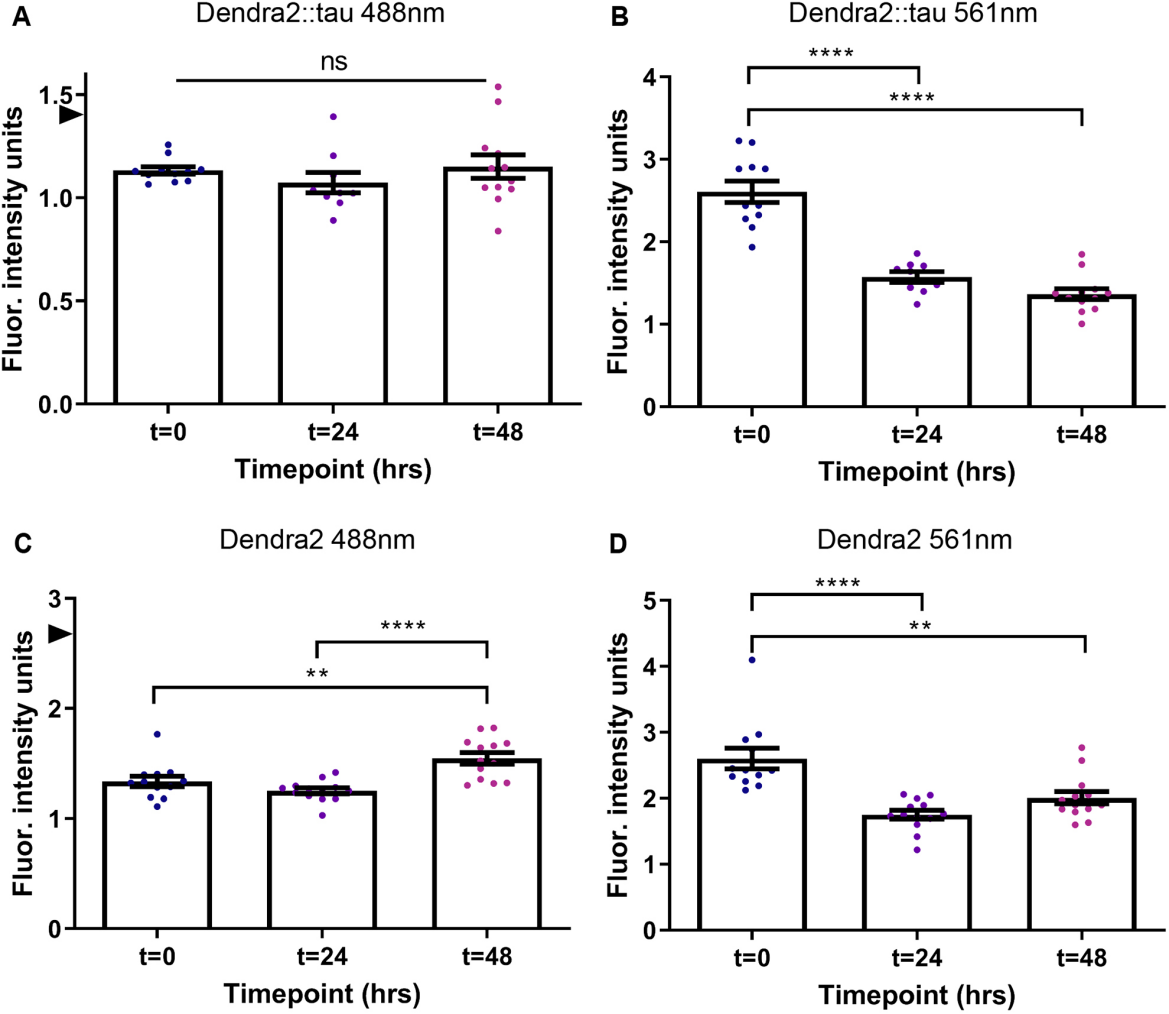
**Dendra2::tau protein turns over faster than Dendra2.** (A,B) Mean fluorescence intensity of Dendra2::tau Tg M4 strain head neurons from 488 nm excitation (A) [one-way ANOVA with Tukey's multiple comparison test (*P*=0.4850)] and 561 nm excitation (B) [one-way ANOVA with Tukey's multiple comparison test (*P*<0.0001)]. (C,D) Mean fluorescence intensity of Dendra2-only strain head neurons from 488 nm excitation (C) [one-way ANOVA w/Tukey's multiple comparison test (*P*<0.0001)] and 561 nm excitation (D) [one-way ANOVA w/ Tukey's multiple comparison test (*P*<0.0001)]. *n*≥9 animals/strain/timepoint. Arrowheads indicate average fluorescence intensity of nonconverted Dendra2::tau (1.42) and Dendra2 (2.69) signal averages in A and C, respectively. The 488 nm wavelength measures non-photoconverted green Dendra2, whereas the 561 nm wavelength measures photoconverted red Dendra2. Representative images are shown in Fig. S3. Data are mean±s.e.m. ns, not significant; **P<0.01; ****P<0.0001.

Day 1 adult Dendra2 and Dendra2::tau Tg M4 strains were photoconverted and serially imaged over 48 h (Fig. 3; Fig. S3). Tg M4 was selected for this experiment owing to its intermediate level of Dendra2::tau expression compared to that in other strains and the feasibility of imaging with the same optical configuration as the Dendra2 control strain.

Dendra2::tau and Dendra2 green fluorescence decreased upon photoconversion and never returned to initial intensity levels (Fig. 3A,C), replicating earlier studies (Hamer et al., 2010; Bolková and Lanctôt, 2016). We found that photoconverted Dendra2::tau red fluorescence decreased by 47.7% over 48 h, whereas photoconverted Dendra2 red fluorescence decreased by 22.9% during the same time (Fig. 3B,D), indicating that Dendra2::tau protein turnover rate is higher than that of Dendra2 alone. The relative stability of photoconverted Dendra2 alone recapitulates that observed in previous studies (Zhang et al., 2007; Hamer et al., 2010).

### Dendra2::tau protein expression level affects its degradation rate

**Fig. 4.**
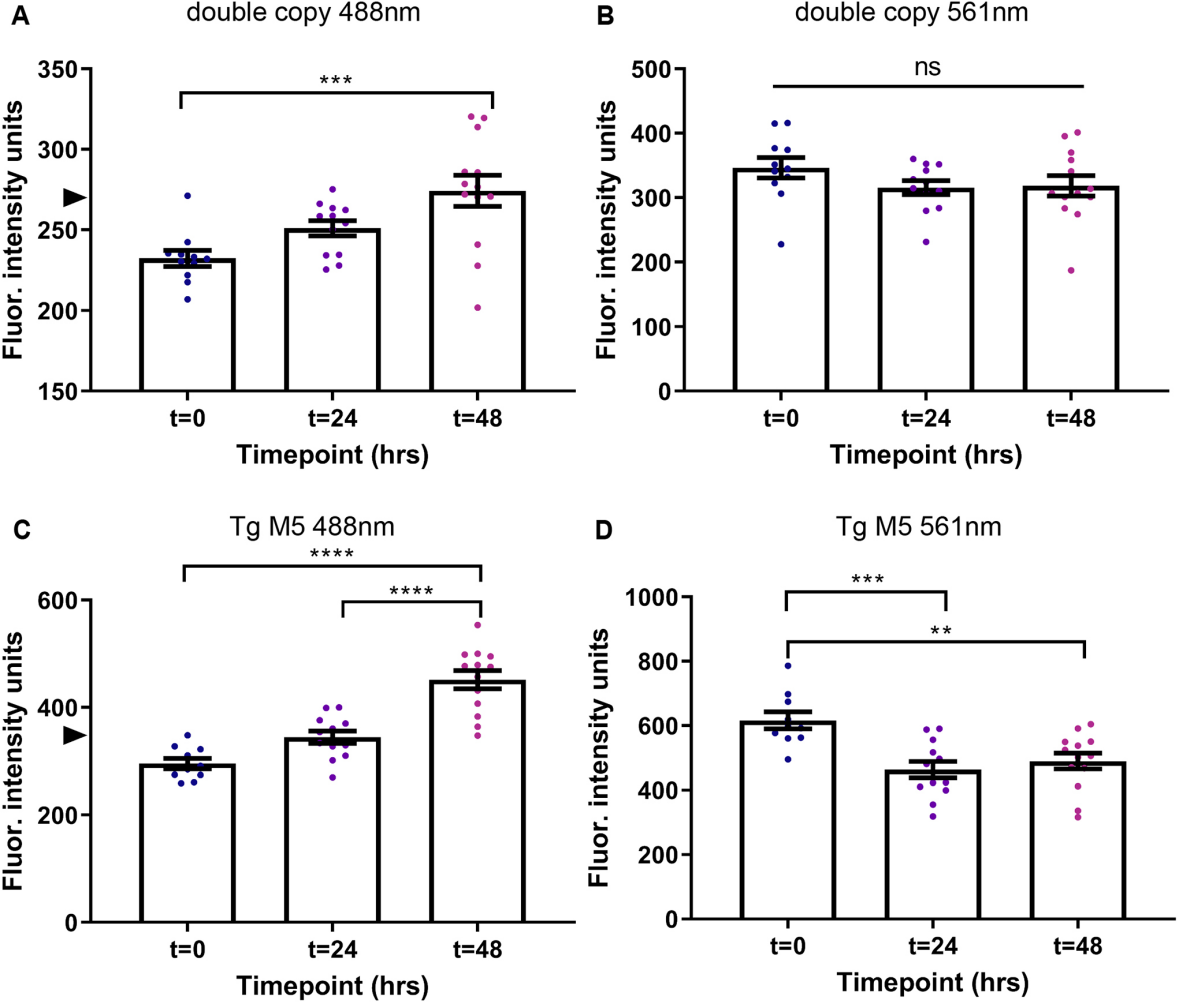
**Dendra2::tau protein turnover depends on expression level.** (A,B) Mean fluorescence intensity of Dendra2::tau double-copy strain head neurons from 488 nm excitation (A) [one-way ANOVA with Tukey's multiple comparison test (*P*=0.0009)] and 561 nm excitation (B) [one-way ANOVA with Tukey's multiple comparison test (*P*=0.2799)]. (C,D) Mean fluorescence intensity of Dendra2::tau Tg M5 strain head neurons from 488 nm excitation (C) [one-way ANOVA with Tukey's multiple comparison test (*P*<0.0001)] and 561 nm excitation (D) [one-way ANOVA with Tukey's multiple comparison test (*P*=0.0006)]. *n*≥10 animals/strain/timepoint. Arrowheads indicate average fluorescence intensity of nonconverted Dendra2::tau in double-copy (269.29) and Tg M5 (351.57) strains in A and C, respectively. The 488 nm wavelength measures non-photoconverted green Dendra2, whereas the 561 nm wavelength measures photoconverted red Dendra2. Representative images are shown in Fig. S4. Data are mean±s.e.m. ns, not significant; **P<0.01; ***P<0.001; ****P<0.0001.

A double-transgenic strain, hereafter referred to as ‘double-copy’ strain, was generated by crossing the two single-copy strains Tg S1 and Tg S3, resulting in greater Dendra2 fluorescence that enabled direct comparison with the low-expression multicopy strain Tg M5 (Fig. S4). To determine whether the Dendra2::tau turnover rate changes with the expression level of the protein, the double-copy and Tg M5 strains were photoconverted and serially imaged over 48 h. Although newly synthesized green Dendra2::tau levels increased over 48 h in both strains (Fig. 4A,C), the turnover of photoconverted red Dendra2::tau differed between strains. The double-copy strain failed to exhibit significant turnover of Dendra2::tau over 48 h, whereas the Tg M5 strain demonstrated significant degradation of the protein, resulting in a 20.5% decrease over 48 h (Fig. 4B,D).

Our findings indicate that lower Dendra2::tau expression results in less turnover over 48 h. Although the complete lack of turnover in the double-copy strain may be surprising, it is possible that the amount of Dendra2::tau in this strain is insufficient stress to activate neuronal protein degradative pathways, resulting in no clearance of the protein.

### TDP-43 enhances the Dendra2::tau and Dendra2 phenotype but does not alter Dendra2::tau protein turnover

**Fig. 5.**
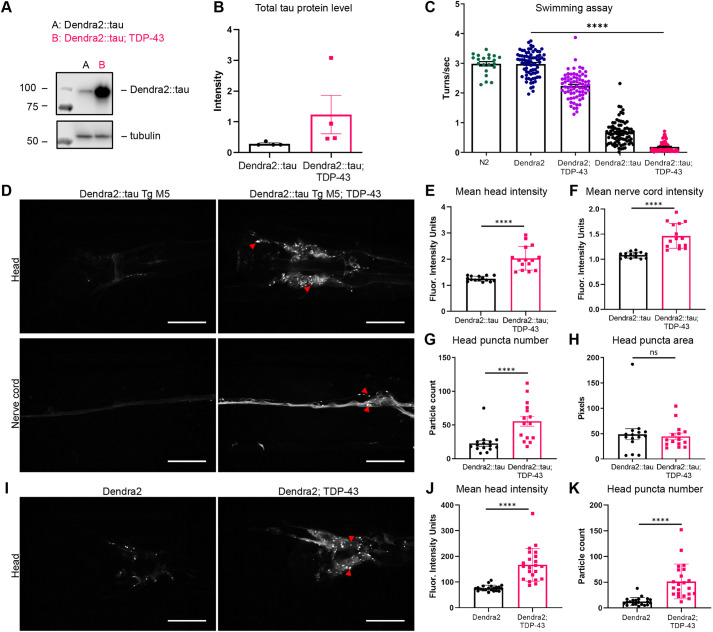
**TDP-43 promotes accumulation of Dendra2::tau and Dendra2 alone.** (A) Representative Western blot detecting total tau in the Dendra2::tau Tg M5 strain alone and with the TDP-43 (CK1943) background. (B) Quantification of the Dendra2::tau band intensity in A normalized to tubulin band intensity [four biological replicates; one-tailed paired *t*-test (*P*=0.1039)]. (C) Swimming assay [four biological replicates; *n*≥72 animals/strain; *n*=21 for N2; one-way ANOVA with Tukey's multiple comparison test (*P*<0.0001)]. (D) Representative confocal images of head and nerve cord of Dendra2::tau Tg M5 strain alone and with the TDP-43 (CK1943) background. Images have been adjusted with +40% contrast and +40% brightness. (E,F) Quantification of mean fluorescence intensity as shown in D of the head (*P*<0.0001) and nerve cord (*P*<0.0001) (*n*≥14 animals; unpaired two-tailed *t*-test with Welch's correction). (G,H) Quantification of mean puncta number (*P*=0.0008) and puncta area (*P*=0.7357) in head images as shown in D (*n*=15 animals; unpaired two-tailed *t*-test with Welch's correction). (I) Representative confocal images of the head of strains expressing Dendra2 alone and Dendra2 in the TDP-43 (CK1943) background. Images have been adjusted with +40% contrast and +40% brightness. (J,K) Quantification of mean puncta number (*P*<0.0001) and puncta area (*P*<0.0001) in head images as shown in I (*n*≥21 animals; unpaired two-tailed *t*-test with Welch's correction). Red arrowheads in representative images indicate puncta. Scale bars: 25 μm. Data are mean±s.e.m. ns, not significant; ****P<0.0001.

Our group previously showed strong enhancement of tau pathology in *C. elegans* transgenic for untagged WT human tau by WT human TDP-43 (Latimer et al., 2022). The tau strain exhibited a mild disease phenotype alone but a moderate to strong disease phenotype with TDP-43, a phenomenon recapitulated in Dendra2::tau-expressing *C. elegans* (Fig. 5A-C). We crossed a strain with low expression of WT human TDP-43 (CK1943) with the Dendra2::tau Tg M5 strain, which expresses the lowest level of Dendra2::tau among the multicopy strains. Tg M5 was chosen for this experiment because higher expression of tau compromises viability in combination with the *TDP-43* transgene. Co-expression of TDP-43 with Dendra2::tau increased tau protein accumulation, exacerbated motor deficits and enhanced fluorescence intensity (Fig. 5). Dendra2::tau; TDP-43 animals showed increased fluorescence intensity in the head and nerve cord (Fig. 5D-F) as well as increased puncta number (Fig. 5G) but no change in puncta area in the head (Fig. 5H). Nerve cord puncta could not be quantified owing to high background signals. These data suggest that TDP-43 drives Dendra2::tau accumulation or impairs its degradation and promotes the aggregation of Dendra2::tau into visible puncta. Furthermore, TDP-43 enhancement of Dendra2::tau was observed in the single-copy Dendra2::tau strain Tg S1 over the course of aging (Fig. S1).

Surprisingly, TDP-43 increased the fluorescence intensity and puncta number in the head of Dendra2-only animals (Fig. 5I-K) and decreased motility (Fig. 5C), but not to the same extent as for Dendra2::tau-expressing animals. Therefore, we suspect that TDP-43 impairs general proteostasis of Dendra2 as a long-lived protein in the neuron, as it has been shown that TDP-43 perturbs various proteostasis mechanisms (Leibiger et al., 2018; Ormeño et al., 2020; de Mena et al., 2021; Yin et al., 2021).

**Fig. 6.**
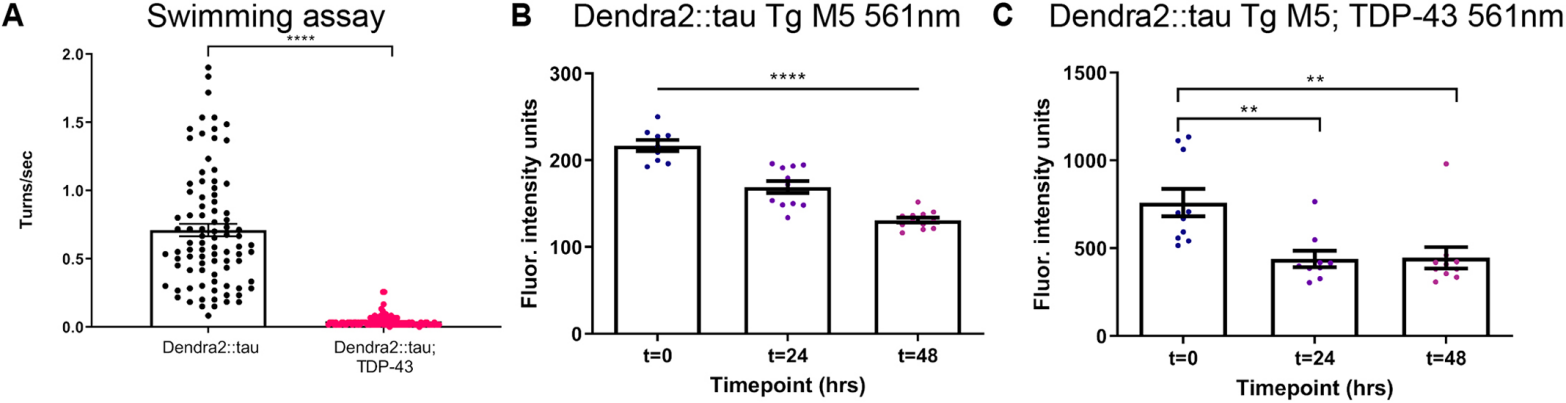
**TDP-43 does not affect Dendra2::tau protein turnover.** (A) Swimming assay of the Dendra2::tau Tg M5 strain alone and with the TDP-43 (CK402) background [three biological replicates; *n*≥88 animals/strain; unpaired two-tailed *t*-test (*P*<0.0001)]. (B) Mean fluorescence intensity of Dendra2::tau Tg M5 strain head neurons from 561 nm wavelength excitation [one-way ANOVA with Tukey's multiple comparison test (*P*<0.0001)]. (C) Mean fluorescence intensity of Dendra2::tau Tg M5; TDP-43 (CK402) strain head neurons from 561 nm wavelength excitation [one-way ANOVA with Tukey's multiple comparison test (*P*=0.0017)]. *n*≥9 animals/strain/timepoint. The 561 nm wavelength measures photoconverted red Dendra2. Representative images are shown in Fig. S5. Data are mean±s.e.m. **P<0.01; ****P<0.0001.

To determine whether TDP-43 affects Dendra2::tau protein turnover, we generated a new Dendra2::tau Tg M5; TDP-43 strain using a different strain with low expression of WT human TDP-43 (CK402) with a fluorescent reporter compatible with acquisition of photoconverted red Dendra2::tau. This new Dendra2::tau Tg M5; TDP-43 strain also exhibited behavioral impairment in the swimming assay compared to Tg M5 alone (Fig. 6A). Upon photoconversion, the Dendra2::tau Tg M5 and Dendra2::tau Tg M5; TDP-43 strains exhibited a similar proportion of Dendra2::tau turnover (39.6% versus 41.4% decrease, respectively) over 48 h (Fig. 6B,C). These results indicate that although TDP-43 increases Dendra2::tau accumulation, it does not impair Dendra2::tau degradation.

### Suppressors of tauopathy differentially interact with Dendra2::tau

**Fig. 7.**
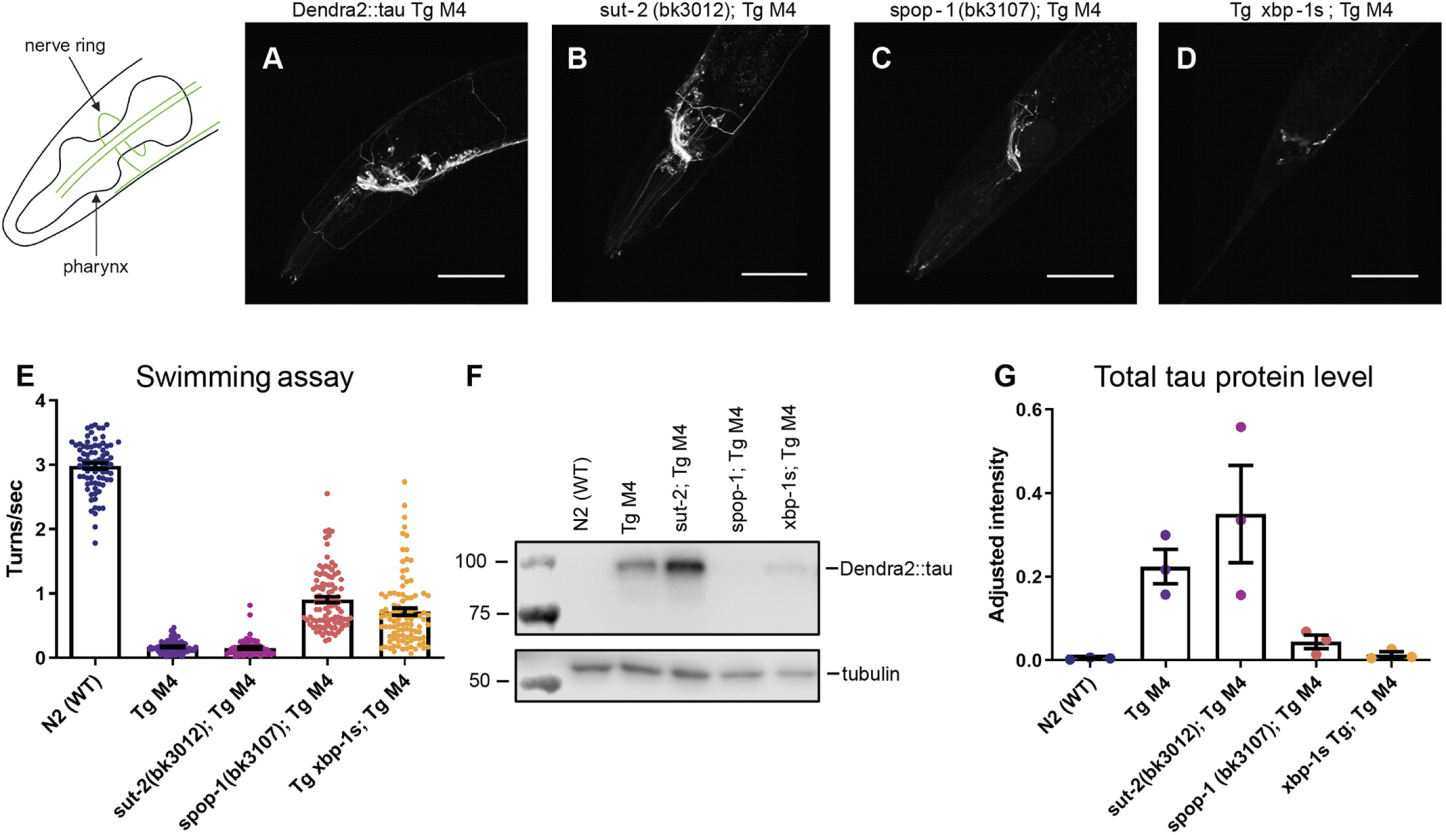
**Differential suppression of Dendra2::tau by known suppressors of tauopathy.** (A-D) Schematic (left) and head images (A-C) of suppressors of tauopathy in the Dendra2::tau Tg M4 background. The tail is shown for Tg *xbp-1s* (D) due to the fluorescent pharyngeal marker. Images have been adjusted with +40% contrast and +40% brightness. Scale bars: 50 μm. (E) Swimming assay [four biological replicates; *n*≥63 animals; one-way ANOVA with Tukey's multiple comparison test (*P*<0.0001)]. (F) Western blot of total tau. (G) Quantification of the Dendra2::tau band intensity in F normalized to tubulin band intensity [three biological replicates; one-way ANOVA with Tukey's multiple comparison test (*P*=0.0045)]. Data are mean±s.e.m.

We crossed known genetic suppressors of tauopathy (sut genes) into the moderately expressing Dendra2::tau strain Tg M4, which displays a strong behavioral and fluorescence phenotype for detection of genetic suppression (Fig. 7A). We expected to observe levels of Dendra2::tau suppression similar to previous findings in untagged tau backgrounds (Table 1). However, we found that *sut-2* did not suppress Dendra2::tau phenotypes tested, including fluorescence intensity, motility or tau protein accumulation (Fig. 7B,E-G). Interestingly, although *spop-1* suppressed the motility defects of the Dendra2::tau Tg M4 strain more strongly than *xbp-1s* (Fig. 7E), both *spop-1* and *xbp-1s* suppressed accumulation of Dendra2::tau to similarly low levels (Fig. 7C,D,F,G). In summary, not all sut genes shown to suppress tauopathy phenotypes driven by untagged tau suppressed the tauopathy phenotype in the Dendra2::tau model. Additionally, for any given sut gene, the degree of suppression of Dendra2::tau accumulation will not necessarily be proportional to the change in motility deficits.

**Table 1.**
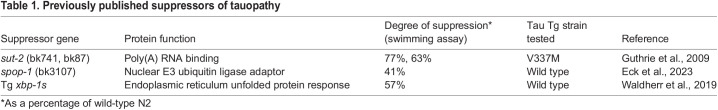
Previously published suppressors of tauopathy

## DISCUSSION

In AD and other tauopathies, neurons must clear pathological tau to maintain neuronal health, and failure to do so results in tau aggregation, neuronal dysfunction and neurodegeneration. Understanding regulation of tau proteostasis is key to developing treatments that can modulate tau accumulation and clearance in disease. Investigation of tau proteostasis mechanisms would benefit from the capacity to visualize tau *in vivo* in a genetically tractable model organism in which mammalian proteostasis pathways are conserved.

We present Dendra2::tau transgenic *C. elegans* as a useful tool for modeling tau proteostasis. We found that, congruent with untagged tau models, Dendra2-tagged tau burden correlated positively with phenotype severity in multicopy Dendra2::tau Tg strains. In contrast, single-copy Dendra2::tau Tg strains lacked obvious disease-related phenotypes owing to low tau levels. The lack of a distinct disease phenotype in this single-copy model indicates the usefulness of multicopy arrays in modeling disease in *C. elegans*. Given the known high levels of neuronal tau occurring *in vivo* in disease states and the low-level tau expressed from single-copy transgenic strains, the single-copy strategy appears to be poorly suited for modeling tauopathy but could be suitable for identifying genetic enhancers and pathways contributing to tau proteostasis.

Investigating the effects of known tau suppressors on the Dendra2::tau model revealed interesting results. The sut genes *spop-1* and *xbp-1s* suppressed Dendra2::tau fluorescence, accumulation and motility similarly to previously published effects on untagged tau, whereas *sut-2* failed to suppress it at all. These discrepancies may be due to disrupted protein–protein interactions and could be used to help elucidate the mechanism by which each sut gene suppresses the tau phenotype. A known enhancer of tauopathy, WT human TDP-43, enhanced the fluorescence and behavioral phenotype of a low-expressing Dendra2::tau strain. Interestingly, WT human TDP-43 produced a similar effect in a Dendra2-only control strain, suggesting that TDP-43 disrupts general proteostasis in neurons (Leibiger et al., 2018; Xia et al., 2016; Lee et al., 2020; Ormeño et al., 2020; Yin et al., 2021; Keating et al., 2022).

In addition to the abundance and localization of the tagged protein, Dendra2 can be used to determine the turnover rate of the protein of interest by optical pulse labeling. Photoconversion of Dendra2- and Dendra2::tau-expressing animals showed Dendra2::tau turnover to be faster than that of Dendra2 alone. Our results support those of previous publications, which show that Dendra2 is as stable if not more stable than Dendra2::tau (Zhang et al., 2007; Hamer et al., 2010). Small discrepancies between our results and previously published reports can be attributable to differences in expression level, transgene promoter, tau isoform or model organism. Photoconversion in the double-copy Dendra2::tau strain compared to that in the lowest-expression multicopy Tg M5 strain showed that less Dendra2::tau expression resulted in less turnover of the protein, which may be due to insufficient activation of proteostatic pathways by the low level of Dendra2::tau expression in the double-copy strain. Interestingly, TDP-43 did not change the rate of Dendra2::tau degradation. Although we had expected TDP-43 to impair Dendra2::tau turnover as TDP-43 dysregulates proteostatic pathways (Leibiger et al., 2018; Ormeño et al., 2020; Yin et al., 2021), our results suggest that TDP-43 does not stabilize Dendra2::tau but instead promotes its accumulation, possibly through enhanced protein synthesis to enhance tauopathy phenotype. It is also possible that the higher expression of Dendra2::tau protein in the Dendra2::tau Tg M5; TDP-43 strain more strongly activated neuronal proteostatic pathways such as ER stress signaling (Walker et al., 2013; de Mena et al., 2021) to clear Dendra2::tau, compensating for proteostasis impairment by TDP-43. Future investigation will determine which proteostasis pathways – UPS, ALP or UPR – participate in TDP-43 enhancement of Dendra2::tau alone.

*C. elegans* is an ideal organism for *in vivo* visualization of fluorescently tagged proteins owing to its optical transparency. Recently, Nunez et al. (2022) presented a GFP-tagged tau *C. elegans* model that differs substantially from our Dendra2::tau model in that it (1) is expressed under the *rgef-1* promoter, (2) fuses GFP to the C-terminus of tau, (3) lacks a disease phenotype without aging and (4) lacks visible tau aggregates. Since the initial description of Dendra2 as a photoconvertible protein in 2006 (Gurskaya et al., 2006), Guha et al. (2020) are the only other group to use Dendra2::tau as a probe for studying tau function and pathogenesis in *C. elegans*. In contrast to our multicopy and single-copy pan-neuronal snb-1::Dendra2::1N4R WT tau models, Guha et al. (2020) employed a single copy of mec-7::Dendra2::0N4R tau with phosphomimetic, phospho-ablative or acetylation-mimetic mutations. In this mechanosensory model, Dendra2 expressed by itself exhibits greater fluorescence compared to that of Dendra2::tau, in agreement with our pan-neuronal model. Our Dendra2 strain may have integrated a higher copy number of the Dendra2 construct compared to the Dendra2::tau strains because Dendra2 is putatively less toxic than tau, and this higher copy number could drive increased Dendra2 expression. However, Guha et al. (2020) used a single-copy model and still observed a similar discrepancy in fluorescence between Dendra2 and Dendra2::tau, indicating that the fusion of tau to Dendra2 might interfere with fluorophore maturation or excitation. Interestingly, Guha et al. (2020) did not take advantage of the photoconvertible utility of Dendra2. It is also possible that they encountered the limitations that we describe below.

There are several previously noted limitations to using Dendra2 to visualize and monitor a protein of interest (Pigazzini and Kirstein, 2020). It is most often used as an exogenous overexpression construct, such that the protein of interest and Dendra2 are not expressed at normal physiological levels in the organism. Furthermore, the rate of Dendra2 degradation could affect the degradation rate of the fused protein of interest. Dendra2 has been shown to be highly stable and, in our model, that could mean enhanced stability for tau due to Dendra2 fusion or vice versa. In contrast, Dendra2 cannot be used to monitor proteins with fast turnover rates because of the time required for photoconversion and fluorophore maturation. The optimal wavelength for photoconversion is 405 nm, which happens to be toxic to *C. elegans*; while Dendra2 can also be photoconverted by 488 nm wavelength light, this means that intensive imaging in the 488 nm channel can also drive photoconversion (Bolková and Lanctôt, 2016). Indeed, we observed this phenomenon when attempting fluorescence recovery after photobleaching (FRAP), which measures diffusion of Dendra2::tau into a photobleached region of interest. This indicates that other, more stable, non-photoconvertible eGFP derivatives may be more suitable fusion partners for tau FRAP experiments than Dendra2.

Dendra2 also posed several challenges that complicated its use as a photoconvertible protein for optical pulse-labeling experiments. First, it was impossible to achieve 100% efficiency of photoconversion. Li et al. (2011) reported a photoconversion efficiency of >60% and, to the best of our knowledge, no group that has explicitly reported their data has demonstrated anywhere near 100% efficiency. Although the lack of complete photoconversion does not preclude optical pulse-labeling experiments, the Dendra2 protein also exhibited incomplete turnover over a measurable time frame of 48 h. Dendra2 by itself is extremely stable after photoconversion for 200 min (Zhang et al., 2007) and 24 h (Hamer et al., 2010), making the Dendra2-only model a control with limited utility when trying to assess the stability of a Dendra2-fused protein. Furthermore, no Dendra2-fused proteins reported thus far have exhibited 100% turnover in the measured time frame. For instance, Hamer et al. (2010) show ∼30% (12 h) and ∼50% (24 h) degradation of whole-organism Dendra2 red fluorescence in their UbG76V-Dendra2 *C. elegans* model. Pigazzini and Kirstein (2020) also showed lack of complete turnover of huntingtin-Dendra2 in a single *C. elegans* neuron at 2 h and 24 h post photoconversion. Lopez et al. (2022) demonstrated up to 80% reduction of Dendra2::tau and Dendra2–α-synuclein (SNCA) in a zebrafish neuron 48 h post photoconversion. Specifically, Croft et al. (2021) showed that Dendra2 and Dendra2::tau half-lives are on the order of days in brain-slice cultures.

Overall, we have shown that the Dendra2::tau model allows immediate visualization of tau localization and abundance. We have developed a suite of Dendra2::tau strains with varying degrees of disease severity suitable for either detection of tauopathy suppression or enhancement. By photoconverting Dendra2, we demonstrated faster turnover of Dendra2 fused to tau compared to that of Dendra2 alone, and determined the effect of Dendra2::tau expression level and TDP-43 on Dendra2::tau turnover. This model could expedite the screening process for genetic modifiers of tau accumulation and turnover rate. The Dendra2::tau model allows faster and easier investigation of tau proteostasis and potential identification of pathways that ameliorate tau accumulation and/or promote clearance, which could provide previously unreported molecular targets for treatment of tauopathy.

## MATERIALS AND METHODS

### *C. elegans* strains and maintenance

Worms were maintained at 20°C on nematode growth medium (NGM) plates seeded with OP50 *Escherichia coli* according to standards described by Brenner (1974). For protein extraction, worms were grown on NGM plates containing medium with 5× peptone (PEP), nutrient-rich media for robust *C. elegans* growth, seeded with OP50 (Brenner, 1974). Transgenic strains were engineered by microinjection into the WT N2 strain using an Eclipse TE300 microscope (Nikon, Tokyo, Japan) and FemtoJet injection rig (Eppendorf, Hamburg, Germany). Multicopy transgenic strains were generated using a conventional transgenic array and genomically integrated by a sub-lethal dose of ultraviolet irradiation from a Stratalinker UV 1800 Crosslinker (Stratagene, Santa Clara, CA, USA). Single-copy Dendra2::tau strains were generated by dual-component recombinase-mediated cassette exchange (Nonet, 2020). All strains were outcrossed to N2 at least twice. Details of strains used are provided in Table S1.

### Protein extraction

Protein extraction and western blotting procedures were conducted as previously described (Kow et al., 2018). Briefly, to create staged populations, worms were grown at 20°C on 150 mm 5× PEP plates to generate populations for hypochlorite treatment for harvest of eggs. Harvested eggs were deposited onto 5× PEP plates and maintained at 20°C for 3 days until worms were harvested from plates using M9 buffer. Worms were pelleted by centrifugation (3000 ***g*** for 45 s) and pellets were subsequently washed three times with 5 ml M9 buffer and transferred to Eppendorf tubes. The buffer was aspirated from centrifuged worms, and pellets were snap frozen with liquid nitrogen prior to storage at −70°C.

Whole-worm protein lysates were created as follows. Worm pellets were thawed on ice and weighed to determine the pellet mass. SDS protein sample buffer (0.046 M Tris, 0.005 M EDTA, 0.2 M dithiothreitol, 50% sucrose, 5% SDS, 0.05% bromophenol blue, 1× concentration) was added to the pellets at a volume (μl) four times the pellet weight (mg). Pellets were sonicated three times, 15 s each at 30% amplitude, returning to ice between sonication sessions. Samples were boiled at 95°C for 10 min and then centrifuged at 13,200 ***g*** for 1 min. Samples were returned to ice prior to gel loading.

### Immunoblotting

For immunoblotting, 5-10 μl of lysate was loaded into each well of a 4-15% pre-cast Criterion SDS-PAGE gradient gel (3450028, Bio-Rad, Hercules, CA, USA). Gels were run at 200 V for 60 min, after which proteins were transferred to polyvinylidene difluoride (PVDF) membranes (Bio-Rad) at 80 V for 30 min and then blocked in 5% milk in PBS directly post transfer. Membranes were incubated with primary antibodies diluted in 5% milk in PBS (blocking solution) overnight with rocking at 4°C and washed three times in PBS with 0.1% Tween (10 min for each wash). They were then subjected to secondary antibody incubation for 2 h rocking at room temperature, washed three times with PBST and detected using a chemiluminescence kit (Bio-Rad, 1705060). Blots were imaged and quantitated using a Odyssey Fc 2800 imager (LI-COR, Lincoln, NE, USA). Details of antibodies used in this study are given in Table S2.

### Motility assays

#### Radial assay

Assessments of *C. elegans* locomotion were carried out as previously described (Currey and Liachko, 2021). In brief, ten to 15 day 1 adult worms were placed at the center of a 100 mm 5× PEP plate. Animals were allowed to move freely for 24 h at 20°C and the radial distance traveled from the start point was recorded.

#### Swimming assay

Day 1 adult worms were obtained by egg lay at 20°C 3 days prior. Worms were moved to the assay room and allowed to acclimate to ambient room temperature for at least 30 min. One strain at a time, worms were washed from NGM plates to food-free 35 mm video plates with 2 ml of M9, allowed to acclimate to M9 buffer for 10 s prior to a 1 min video recording. Videos were acquired using the WormLab platform (MBF Bioscience, VT, USA). After videos were taken, worm movement behavior was analyzed using the WormTracker software (MBF BioScience). Body bends from the mid-point body location of each worm tracked were counted. The total number of body bends was divided by the track length to give the frequency of body bends per second.

### Imaging

Unless otherwise specified, day 1 adult worms were mounted on a 4% agarose gel pad in 50-500 mM sodium azide solution and fixed on coverslips using nail polish and/or molten petroleum jelly. Worms were imaged on a Nikon A1R confocal microscope using 40× oil, 60× oil or 100× oil immersion objectives (Nikon, Melville, NY, USA). Representative images are maximum-intensity projections of *z*-stack images. Analysis was performed using ImageJ Java (Schneider et al., 2012). Corresponding worm diagrams were generated using BioRender.com (Toronto, ON, CA). Representative images in Figs 5 and 7 and Fig. S3 were adjusted with +40% contrast and +40% brightness to enhance visibility.

### Dendra2 photoconversion

A 405 nm wavelength lamp positioned 8 cm above the bench was used to photoconvert a single 35 mm unseeded plate of day 1 adult worms at a time for 8 min to achieve maximum photoconversion while minimizing phototoxicity. Non-photoconverted Dendra2::tau and Dendra2 strains were prepared and imaged alongside photoconverted strains to establish baseline green fluorescence intensity without photoconversion. 61-step *z*-stack images centered and focused on the grinder at 100× magnification were acquired on a Nikon A1R confocal microscope (Nikon, Tokyo, Japan). Non-photoconverted green Dendra2 and Dendra2::tau were imaged with the 488 nm laser, whereas the photoconverted red Dendra2 and Dendra2::tau were imaged with the 561 nm laser. Whole-image fluorescence intensities of each maximum-intensity projection in the green (488 nm) and red (561 nm) channels were quantified using ImageJ for the Dendra2::tau Tg M4 and Dendra2-only photoconversion experiment. To exclude gut autofluorescence detected by the stronger laser power required for acquisition of the double-copy Dendra2::tau and Tg M5 strains, quantification of fluorescence intensity for these experiments were performed on a standardized region of interest capturing the nerve ring but excluding the intestine. Fluorescence intensities at t=24 h and t=48 h were normalized to the average posterior pharyngeal bulb diameter of the day 1 adult cohort imaged at t=0 h to account for neuronal expansion with nematode growth. A normalization factor for each animal was calculated by dividing the posterior pharyngeal bulb diameter of each worm by the average posterior pharyngeal bulb diameter from the t=0 h cohort, measured using ImageJ. The fluorescence intensity of each sample was divided by this normalization factor to produce the normalized fluorescence intensity.

### Statistical analyses

All statistical analyses were performed using GraphPad Prism statistical software (GraphPad Software, La Jolla, CA, USA). Statistical significance was determined using one-way ANOVA with Tukey's multiple-comparison test, one-tailed paired *t*-test or unpaired two-tailed *t*-test with Welch's correction (*P<0.05, **P<0.01, ***P<0.001, ****P<0.0001). The average values reported are the means, with error bars representing standard error of the mean (s.e.m.).

## Supplementary Material

10.1242/dmm.050473_sup1 Supplementary information
