# Supplementary material for: Transgenic Dendra2::tau expression allows in vivo monitoring of tau proteostasis in Caenorhabditis elegans
Source: Dis Model Mech. 2024 Mar 28;17(3):dmm050473. doi: 10.1242/dmm.050473 (PMC10985736; doi:10.1242/dmm.050473)
Supplement: Supplementary information [file dmm-17-050473-s1.pdf]

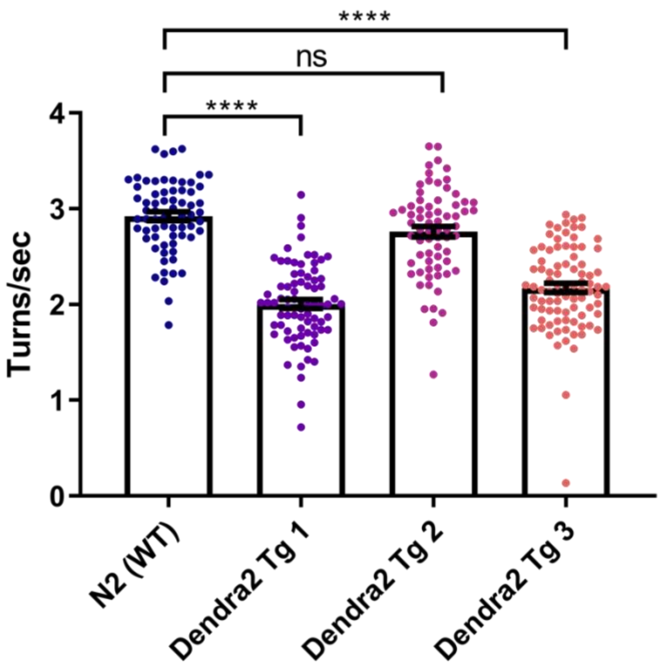

Fig. S1. Dendra2 alone causes minimal neurological impairment measured by swimming assay. [3 biological replicates; N≥67 animals; 1-way ANOVA with Tukey's multiple comparison test ($p<0.0001$)]. Dendra2 Tg 2 strain chosen for experimentation due to no significant difference in swimming performance compared to N2. Error bars: SEM.

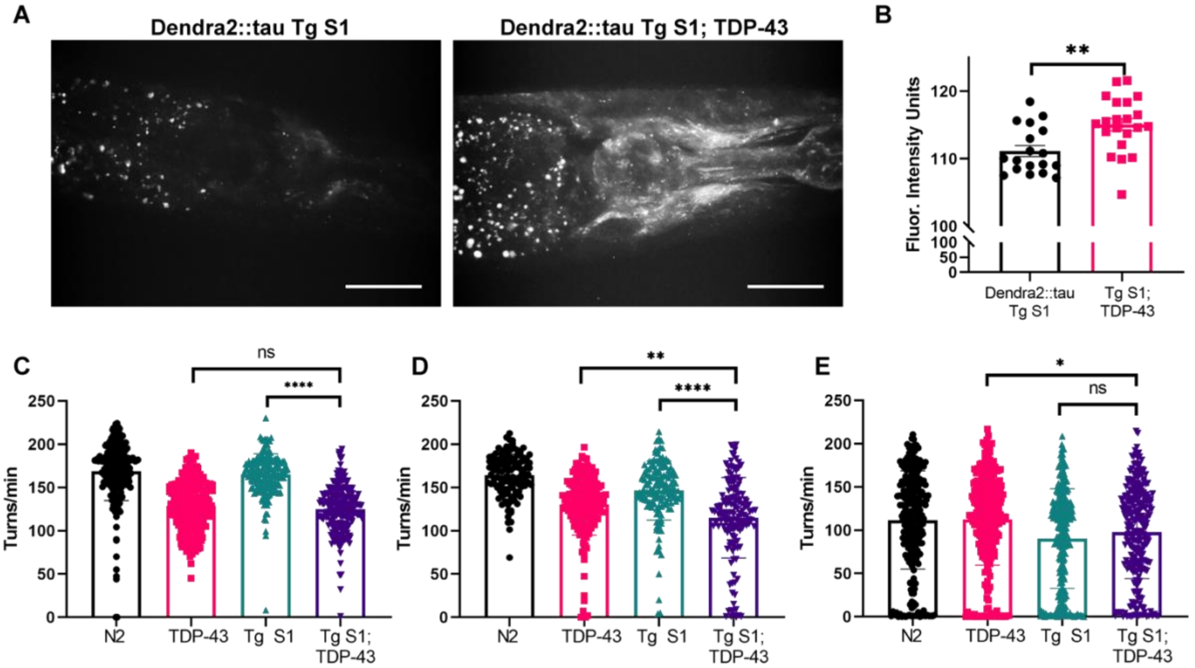

Fig. S2. TDP-43 enhances disease phenotype of single-copy Dendra2::tau *C. elegans*. A Representative images of day one adult single-copy Dendra2::tau Tg S1 with and without wild-type human TDP-43 co-expression. **B** Mean fluorescence intensity of head images from single-copy Dendra2::tau Tg S1 with and without wild-type human TDP-43 co-expression. Swimming assay from **C** day one adults [3 biological replicates; N≥174 animals; 1-way ANOVA with Tukey's multiple comparison test ($p<0.0001$)], **D** day five adults [3 biological replicates; N≥118 animals; 1-way ANOVA with Tukey's multiple comparison test ($p<0.0001$)], and **E** day eight adults [3 biological replicates; N≥247 animals; 1-way ANOVA with Tukey's multiple comparison test ($p<0.0001$)]. Error bars: SEM.

Disease Models & Mechanisms • Supplementary information

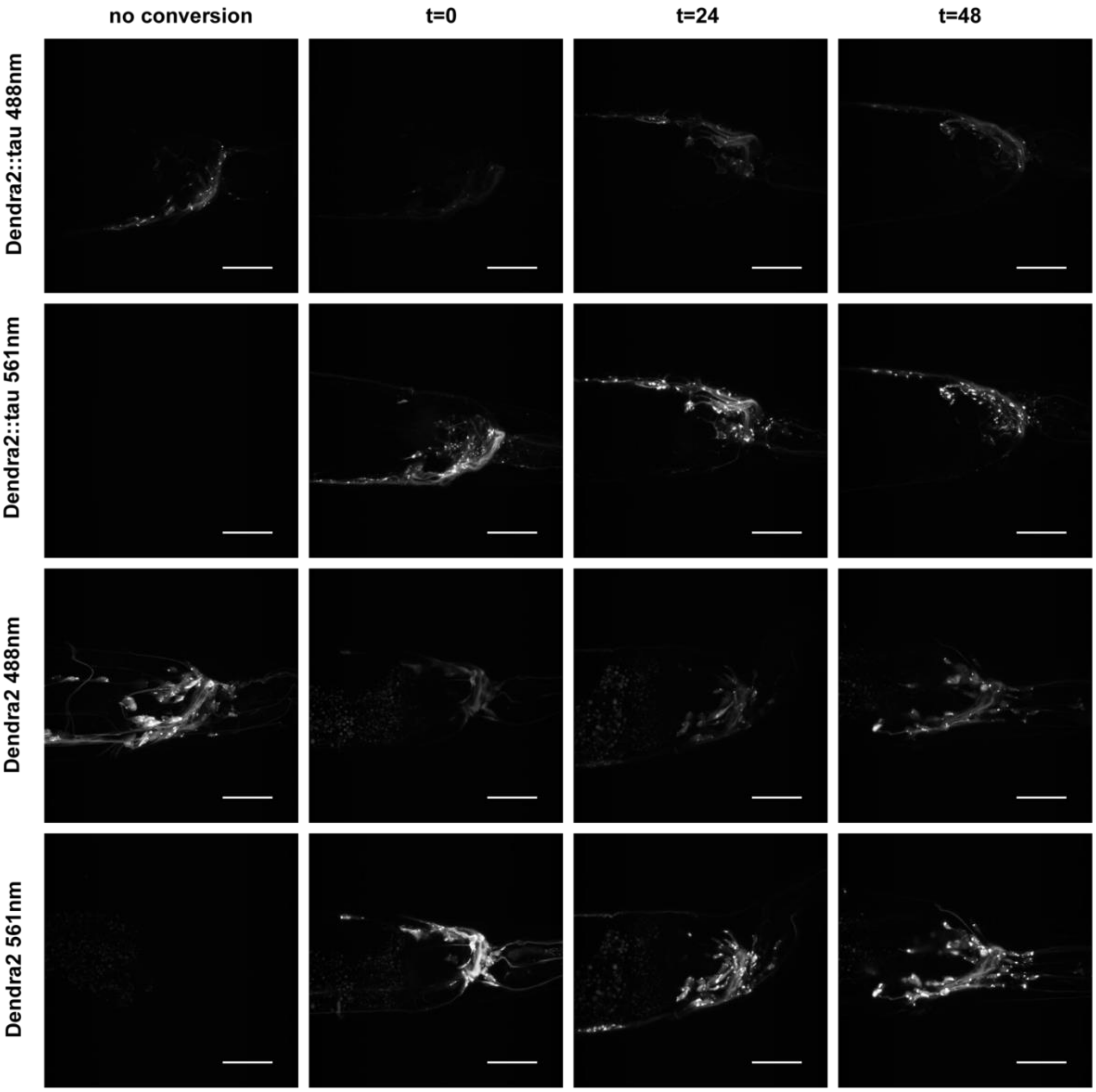

Fig. S3. Representative images of Dendra2::tau Tg M4 and Dendra2 photoconversion. Images adjusted with +40% contrast and +40% brightness. t = time in hours. Scale bar: 25μm.

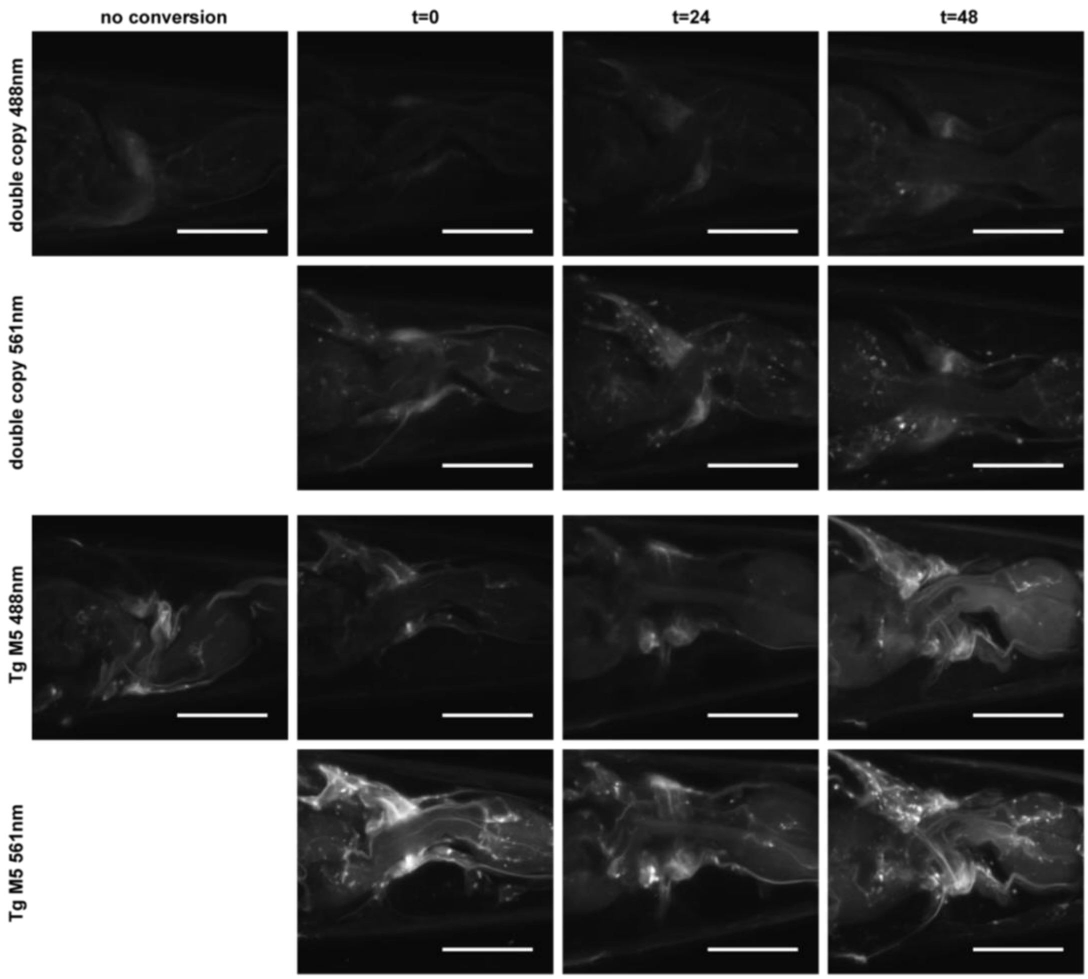

Fig. S4. Representative images of Dendra2::tau double copy and Tg M5 photoconversion. Images cropped to ROI. 561nm channel not acquired for worms that were not photoconverted. t = time in hours. Scale bar: 25μm.

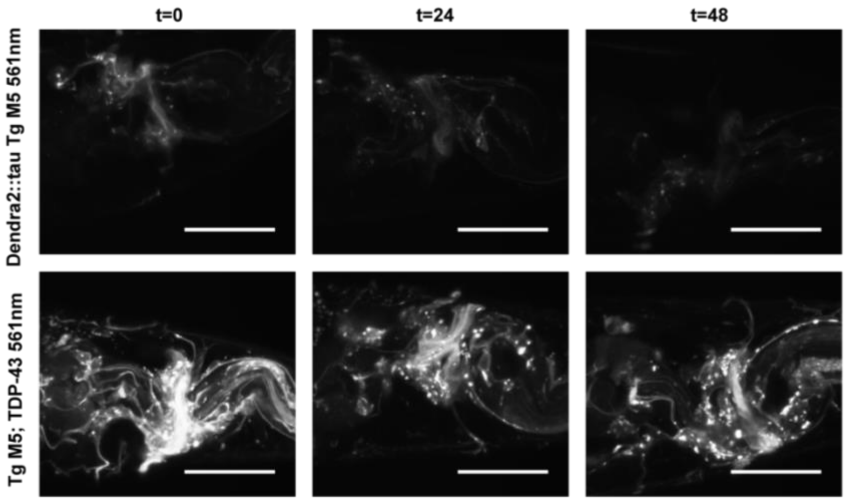

Fig. S5. Representative images of Dendra2::tau Tg M5 and Dendra2::tau Tg M5; TDP- 43 (CK402) photoconversion. 488nm channel not acquired due to green fluorescent body wall reporter of CK402. Images cropped to ROI. t = time in hours. Scale bar: 25µm.

Disease Models & Mechanisms • Supplementary information

Table S1. ***C. elegans*** **strains used in this study.**

Strain	Abbreviation	Genotype	Source
N2	N2 (WT)	Bristol, Great Britain wild-type isolate	CGC* (Brenner, 1974)
CK144	Tau (high)	*bkIs144[Paex-3::Tau WT(1N4R); Pmyo-2::GFP]*	(Kraemer *et al.*, 2003)
CK1441	Tau (low)	*bkIs1441[Paex-3::Tau WT(1N4R);Pmyo-2::dsRED]*	(Benbow *et al.*, 2020)
CK2564	Dendra2 Tg 1	*bkIs2564[Psnb-1::Dendra2]*	This study
CK2565	Dendra2 Tg 2	*bkIs2565[Psnb-1::Dendra2]*	This study
CK2566	Dendra2 Tg 3	*bkIs2566[Psnb-1::Dendra2]*	This study
CK2174	Tg M1	*bkIs2174[Psnb-1::Dendra2::hTau WT (1N4R)]*	This study
CK2175	Tg M2	*bkIs2175[Psnb-1::Dendra2::hTau WT (1N4R)]*	This study
CK2176	Tg M3	*bkIs2176[Psnb-1::Dendra2::hTau WT (1N4R)]*	This study
CK2177	Tg M4	*bkIs2177[Psnb-1::Dendra2::hTau WT (1N4R)]*	This study
CK2178	Tg M5	*bkIs2178[Psnb-1::Dendra2::hTau WT (1N4R)]*	This study
CK2351	Tg S1	*bkIs2351[Psnb-1::Dendra2::hTau WT (1N4R)]*	This study
CK2352	Tg S2	*bkIs2352[Psnb-1::Dendra2::hTau WT (1N4R)]*	This study
CK2353	Tg S3	*bkIs2353[Psnb-1::Dendra2::hTau WT (1N4R)]*	This study
CK2354	Tg S4	*bkIs2354[Psnb-1::Dendra2::hTau WT (1N4R)]*	This study
CK2188	*sut-2;* Tg M4	*sut-2(bk3012)*; *bkIs2177[Psnb-1::Dendra2::hTau WT (1N4R)]*	This study
CK2216	*Tg xbp-1s;* Tg M4	*uthIs270[rab-3p::xbp-1s, myo-2p::tdTomato]; bkIs2177[Psnb-1::Dendra2::hTau WT (1N4R)]*	This study
CK2546	*spop-1;* Tg M4	*spop-1(bk3107)*; *bkIs2177[Psnb-1::Dendra2::hTau WT (1N4R)]*	This study
CK1943	TDP-43	*bkIs1943[Psnb-1::hTDP-43 WT::K4aptazyme::unc-54 3'UTR+Pmyo-3::mCherry]*	(Latimer *et al.*, 2022)
CK2656	TDP-43; Dendra2::tau Tg M5	*bkIs2178[Psnb-1::Dendra2::hTau WT (1N4R)]; bkIs1943[Psnb-1::hTDP-43 WT::K4aptazyme::unc-54 3'UTR+Pmyo-3::mCherry]*	This study
CK402	TDP-43	*bkls402[Psnb-1::TDP-43 WT + Pmyo-3::GFP]*	(Currey and Liachko, 2021)

** CGC: Caenorhabditis Genetics Center.*

Table S2. Antibodies used in this study.

Antigen	Host species	Source	Catalog #/clone	Concentration
Tau	Rabbit	Dako	A0024	1:500,000
β-tubulin	Mouse	Developmental Studies Hybridoma Bank	E7	1:5000
Anti-rabbit HRP (secondary)	Goat	Jackson Immuno Research	111-035-144	1:5000
Anti-mouse HRP (secondary)	Goat	Jackson Immuno Research	111-035-146	1:5000
